# Biological Mechanisms of Psychotherapeutic Change: A Systematic Review of Neuroimmune, Autonomic and Epigenetic Changes Following CBT, Mindfulness Based and Trauma-Focused Therapies

**DOI:** 10.1192/bjo.2026.11143

**Published:** 2026-06-30

**Authors:** Naima Gul, Gaurav Uppal, Maryum Maryum, Kasthuri Padiyan, Betsy Marina Babu

**Affiliations:** 1 Leeds Community Healthcare NHS Trust, Leeds, United Kingdom; 2 Satyam Hospital, Ludhiana, India; 3 Tallaght University Hospital, Dublin, Ireland; 4 MVJ Medical College Research Hospital, Bengalore, India; 5 ELNFT, London, United Kingdom

## Abstract

**Aims:**

There is growing evidence that certain subtypes of major depressive disorder, especially treatment-resistant depression and anhedonia-predominant phenotypes, are marked by both chronic neuroimmune activation and disruptions in reward processing circuitry. These results cast doubt on purely monoaminergic models of depression and highlight the need to comprehend how psychotherapeutic interventions may work through biological systems controlling immune function, stress regulation, and plasticity rather than just suppressing symptoms. Some therapies like Cognitive behavioural therapy, Mindfulness based and Trauma focused therapies have been shown to bring positive neuro immune, autonomic and epigenetic changes in brain.With an emphasis on finding common neurobiological pathways and potential biomarkers of treatment response across psychotherapy modalities, this review attempts to outline the biological correlates and mechanisms of action of psychotherapy.

**Methods:**

To find systematic reviews, meta-analyses, and important longitudinal studies looking at biological changes linked to psychotherapy, a targeted systematic search ofMEDLINE/PubMed and major online publishing platforms was carried out. Studies that used biological measurements at two time points (pre- and post-intervention) were eligible. Four predetermined domains were used to classify the extracted biological outcomes:

1. Neuroimaging techniques, such as task-based and resting-state functional neuroimaging;

2. Physiological and autonomic measures of stress management, especially heart rate variability;

3. Indicators of inflammatory and immune function;

4. DNA methylation alterations linked to treatment response are the main focus of epigenetic markers.

**Results:**

People receiving Cognitive behavioural therapy (CBT) for depression consistently show changes in the fronto-cingulate–limbic circuitry, specifically in the medial prefrontal cortex and ventral anterior cingulate cortex (vACC), according to longitudinal functional magnetic resonance imaging (fMRI) studies. Standardized measures like the Beck Depression Inventory (BDI) show a strong correlation between these brain alterations and clinical improvement. Additionally, convergent reductions in limbic reactivity–most notably decreased right amygdala activation–across a variety of successful depression treatments are suggested by coordinate-based meta-analytic evidence, supporting the existence of shared neural pathways of symptom improvement.

Autonomic biomarkers have also emerged as promising predictors of the effectiveness of psychotherapy, particularly in the treatment of post-traumatic stress disorder (PTSD). Higher levels of high-frequency heart rate variability (HF-HRV) under basal conditions, associated with parasympathetic tone, have been related to the magnitudes of improvements following psychological therapy including CBT.

Available evidence for immune-related consequences is inconsistent. On the one hand, trials of mindfulness-based interventions show consistently that mindfulness may have limited and variable effects on inflammation with extensive methodology variations between studies that use similar populations, biological markers used for immune evaluation, and interventions. There is some promising evidence that trauma-focused psychotherapy may show differences in longitudinal changes in peripheral DNA methylation between responders and non-responders to trauma-focused psychotherapy while targeting immune function, stress pathways, and neuroplasticity pathways.

**Conclusion:**

Within psychotherapeutic approaches, the strongest consistently accumulated biological evidence points to two related psychological constructs:

(i) the fronto-cingulate–limbic neural networks that are involved in emotion regulation and self-reference;

(ii) regulation of stress and immune system functions.

However, the existing evidence base is limited by small sample size, heterogeneous experimental designs and biomarker measurements, and the timing of the biological measurements. Next generation randomized controlled trials of psychotherapy should employ harmonized multimodal protocols of biomarkers to examine the longitudinal patterns of the causal mechanisms and to elucidate the clinically relevant biomarkers of the response to the treatment

